# Primary Hypertension as the Presenting Feature of Laurence-Moon-Bardet-Biedl Syndrome: A Report of Two Children

**DOI:** 10.7759/cureus.12617

**Published:** 2021-01-11

**Authors:** Vijayakumary Thadchanamoorthy, Nadeesha Jayasekara, Kavinda Dayasiri

**Affiliations:** 1 Clinical Sciences Department, Faculty of Health Care Sciences, Eastern University, Batticaloa, LKA; 2 Paediatrics Unit, Teaching Hospital, Batticaloa, LKA; 3 Paediatrics, Base Hospital, Mahaoya, LKA

**Keywords:** autosomal recessive, obesity, hypertension, retinitis pigmentosa, polydactyly, brachydactyly, laurence-moon-bardet-biedl syndrome, hypogonadism

## Abstract

Laurence-Moon-Bardet-Biedl syndrome (LMBBS) is a rare ciliopathic genetic multisystem disorder. It has several primary and secondary clinical features, which include central obesity, retinitis pigmentosa, mental subnormalities, polydactyly, and renal dysfunction. The majority of children may not show all clinical features at birth, and clinical features can develop progressively over the years. The authors report two children who were followed up for obesity at the pediatric clinic in the local hospital and the ophthalmology clinic at the tertiary care center, respectively, without a diagnosis, and were referred for specialist pediatric evaluation of hypertension detected on their routine checkup and for further management. Both children were investigated and found to have satisfied criteria for LMBBS in association with primary hypertension. Both children were diagnosed late as LMBBS at 12 years and four years of their age although both of them had enough features to have a suspicion of this syndrome.

## Introduction

Laurence-Moon-Bardet-Biedl syndrome (LMBBS) is a rare, autosomal, recessive multisystem disease characterized by structural and functional defects of organs and tissue with varied embryonic aberrations [1]. It has been mostly reported in consanguineous marriages and reported to have 21 mutated genes [2-3]. This disorder has phenotypic and genotypic heterogeneity [4]. The syndrome was first described by Laurence and Moon in a seven-year-old girl in 1866, followed by Bardet, in a four-year-old girl in 1920. Finally, Biedl named Laurence-Moon-Bardet-Biedl syndrome in 1922 by adding several more clinical features [2]. The incidence varies from 1:140,000 to 1:160,000 live births in North America and Europe while the incidence has been higher in Kuwait and Newfoundland where it is 1:13,500 and 1:17,500, respectively [5]. The male to female ratio had been 1.3:1 [6]. These children usually show their symptoms in the first decade of life [2]. The primary manifestations of this condition include obesity, male hypogonadism, retinal pigment defect, psychological interruption, polydactyly, and renal dysfunction [2,7]. Other secondary features include hepatic fibrosis, brachydactyly, diabetes mellitus, neurological features like ataxia, poor coordination, clumsiness, developmental delay, speech and language deficit, behavioral traits, facial dysmorphism, short statures, hypermobile joints, early osteoarthritis, and dental abnormalities [7-8]. The clinical diagnosis of LMBBS is made based on four primary criteria alone or three primaries with two secondary criteria [5]. Herein, the authors report two children who were referred to hypertension from the local clinic and, subsequently, diagnosed lately as having LMBBS associated with primary hypertension at the specialist pediatric clinic in the tertiary care center. Although the genetic study is available to detect the involved gene [3,9] and confirm the syndrome we couldn't do due to financial constraints.

## Case presentation

Case history 1

A 12-year-old boy who has been followed up at the eye clinic for poor vision was referred to a specialist pediatric clinic for the evaluation of hypertension. He was the sixth child of consanguineous parents and was born by a lower segment cesarean section due to a large estimated fetal weight. His birth weight was 4.5 kg and neonatal examination had been normal except polydactyly in both hands and feet. He had an accelerated growth while on a normal diet for which he has been followed up at the obesity clinic of the local hospital. When the child started schooling at the age of six years, he had difficulty in reading and suggested to have spectacles for refractory errors. Despite treatment, he had gradual deterioration of vision and sought specialist opinion where he was diagnosed to have bilateral astigmatism with dense amblyopia and possible congenital nystagmus, but there were no features suggestive of retinitis pigmentosa. He was concluded to have nystagmus secondary to poor vision. At 12 years of age, he had early features of retinitis pigmentosa. Further, he had mild global developmental delay and very poor school performance.

**Figure 1:**
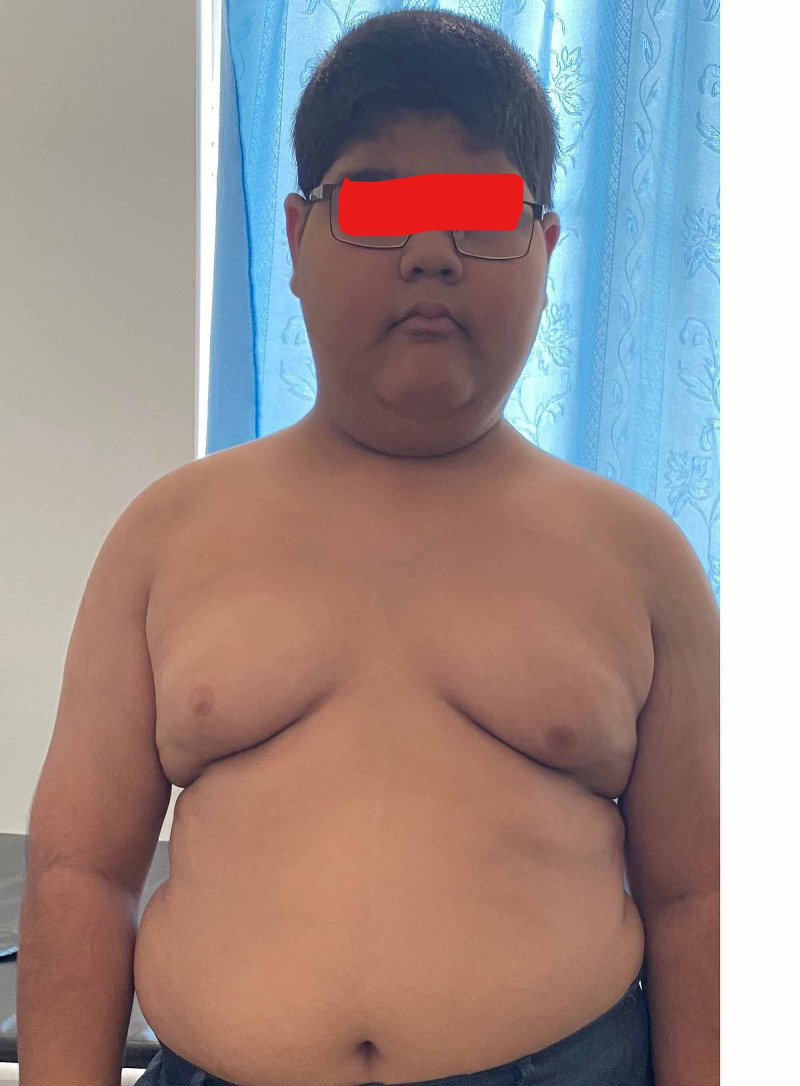
The obese boy shows a round face, double chin, gynecomastia, and pendulous abdomen

**Figure 2:**
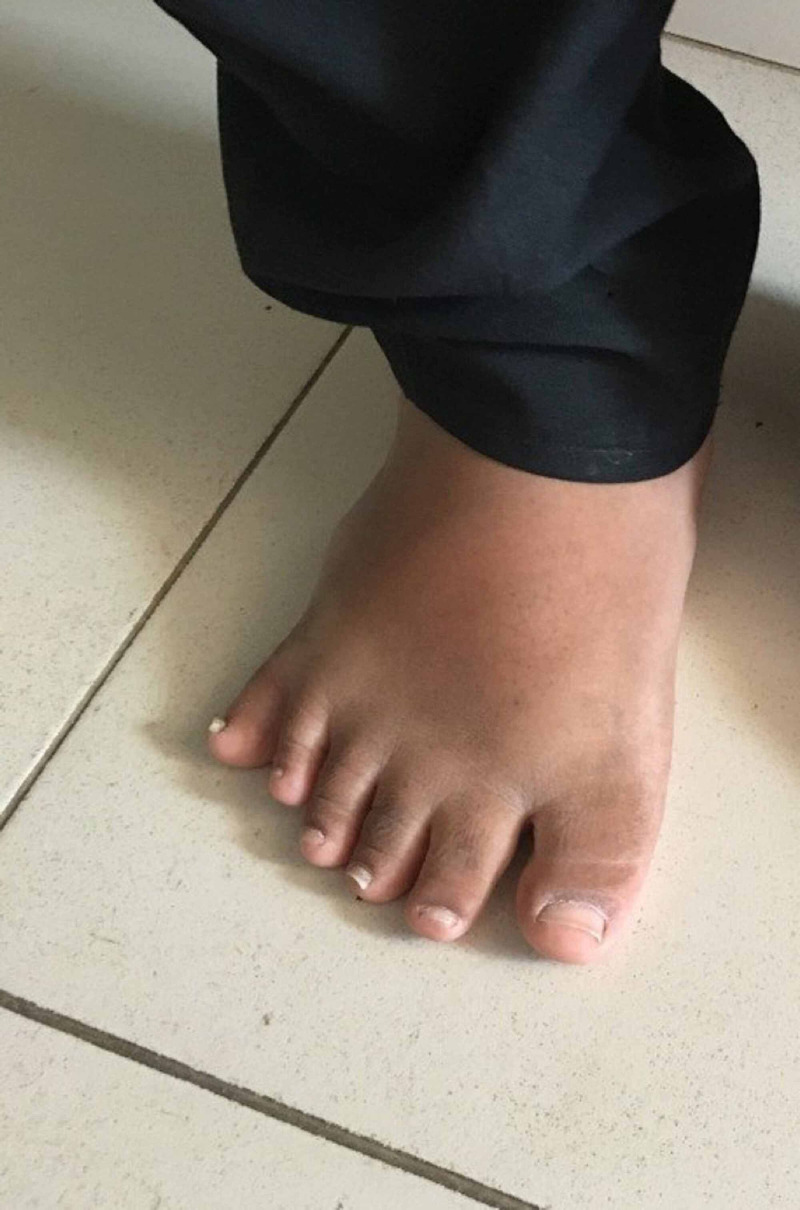
Polydactyly in the foot of the boy

**Figure 3:**
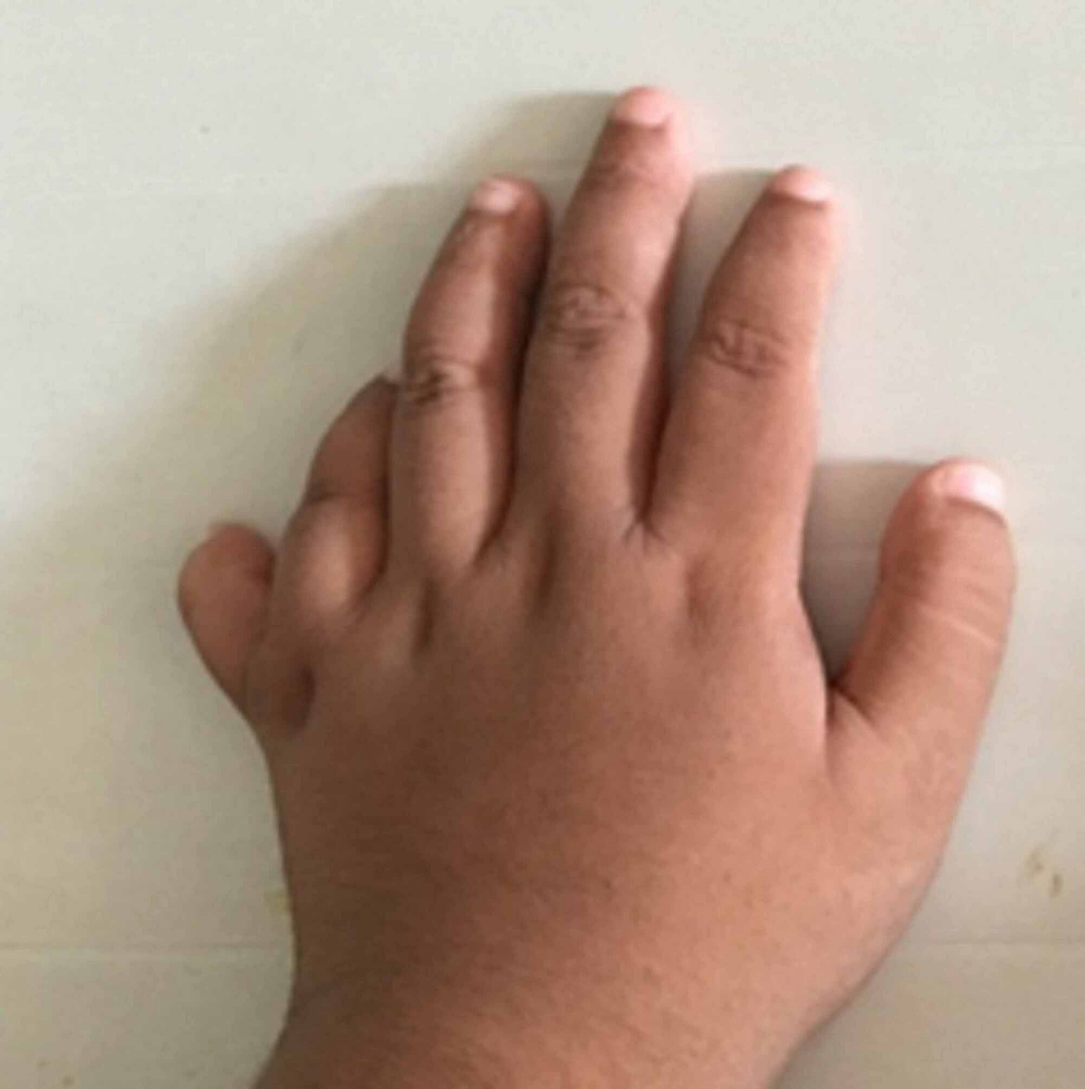
Polydactataly in the hand of the boy

**Figure 4:**
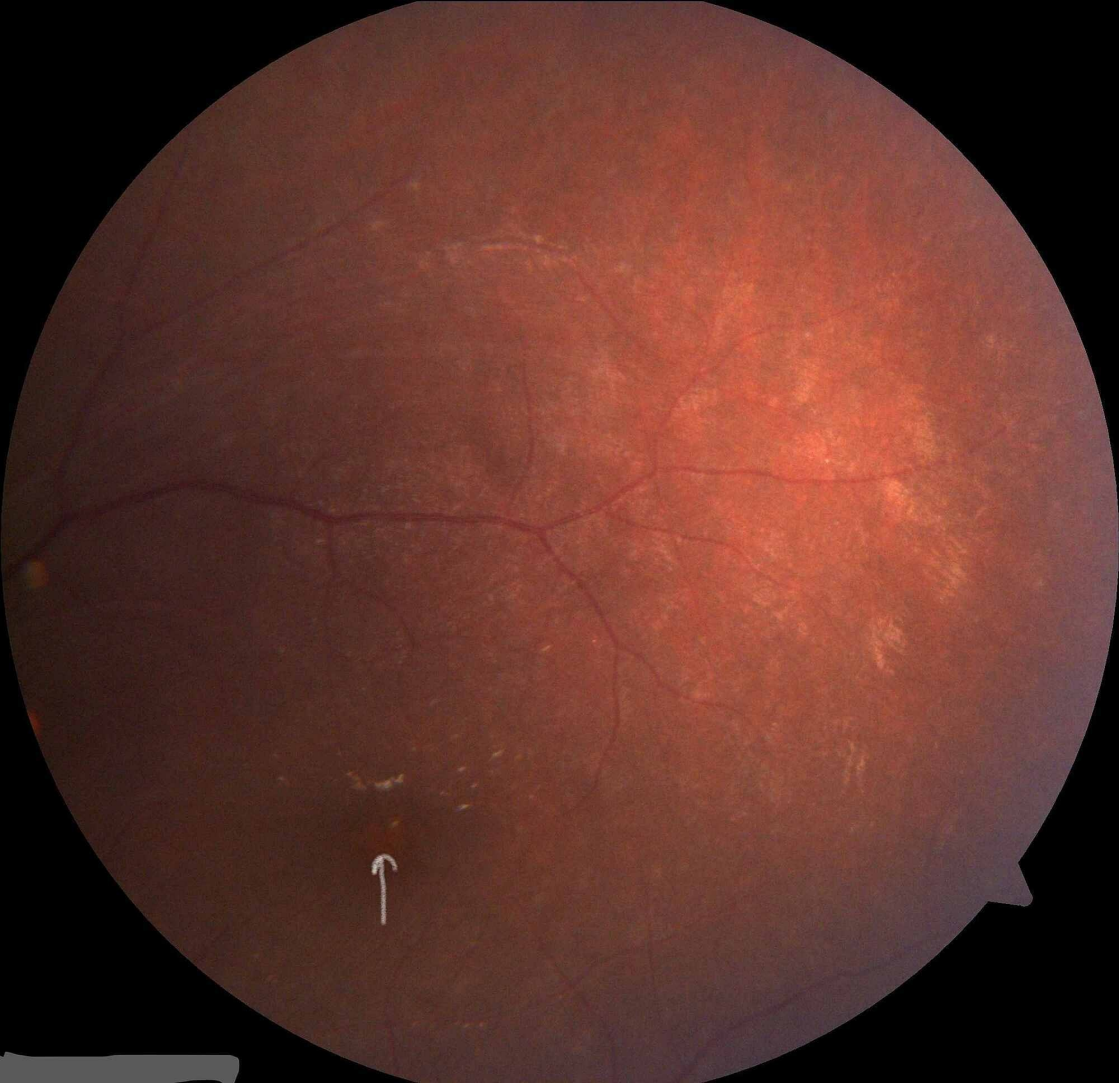
Funduscopic picture shows the attenuation of blood vessels (white arrow) of the 12-year-old boy

Physical examination revealed a rounded face with rowing nystagmus, weight 78.4 kg (>97th centile), height 146 cm (between 10th and 25th centile), and body mass index (BMI) 36.08 kgm^2^ (> 97th centile) (Figure 1). There was postaxial polydactyly with brachydactyly in both hands and feet and broad short feet (Figures 2-3). Neurological examination was normal except for reduced visual acuity of 6/24 in both eyes with early stages of retinitis pigmentosa (Figure 4). His pulse rate was 90 beats/minute with good volume; all the peripheral pulses were palpable; blood pressure was 139/95 mmHg (both systolic and diastolic pressure was above the 95th centile), and he had no murmurs. There was micropenis (3.5 cm in length) with bilateral pre-pubertal-sized testis (2.5 ml). Other systems' examination revealed normal findings. His gait was normal. He was in the pre-pubertal stage.

Investigations revealed normal full blood count, C-reactive protein (CRP), erythrocyte sedimentation rate (ESR), renal function test, fasting blood sugar, liver function test, lipid profile, ultrasound abdomen, electrocardiogram, echocardiogram, and thyroid function. His follicular stimulation hormone (FSH) and luteinizing hormone (LH) were elevated, with low testosterone. The rest of the hormonal assay, including the growth hormone and cortisol stimulation test, was within the normal limit. The chromosomal study confirmed male (XY) sex. Doppler excluded bilateral renal artery stenosis. Pheochromocytoma was excluded by a normal 24-hour urinary normetanephrine level. Renal biopsy showed no abnormalities. Further 24-hour ambulatory blood pressure monitoring confirmed the diagnosis of hypertension.

As this patient met the criteria for the diagnosis of LMBBS (Table 1), the diagnosis of LMBBS with primary hypertension was made. He was managed with antihypertensive medication and follow-up was arranged with the multidisciplinary team (MDT). Further, he was referred to a special school following the educational assessment and planned for hormonal therapy at 14 years of age.

**Table 1:** Comparison of both reported patients with standard criteria Source: [5-8]

Primary Criteria	Population %	Case 1	Case 2
Retinitis pigmentosa	93	+	-
Truncal obesity	72-86	+	+
Polydactyly	72-92	+	+
Learning disability	61	+	+
Hypogonadism	59-98	+	-
Renal abnormalities	53	-	+
Secondary Criteria			
Speech delay/disorders	54-81	+	+
Strabismus/cataracts/astigmatism		+	+
Brachydactyly/syndactyly	6-100/8-95	+	+
Developmental delay	50-90	+	+
Nephrogenic diabetes insipidus		-	-
Diabetes mellitus	6-48	-	-
Ataxia/imbalance/poor coordination	40-86	-	-
Hepatic fibrosis		-	-
Anosmia/hyposmia	60		
Dental abnormalities, high arch palate	51	-	-
Left ventricular hypertrophy/congenital heart disease	7	-	-

Case history 2

A four-year-old girl who has been followed up for obesity at the local clinic was referred to the tertiary care hospital for the further evaluation of hypertension detected during a routine checkup. She is the second child born to non-consanguineous parents. She was born by spontaneous vaginal delivery at term and her birth weight was 2.95 kg. Her neonatal examination had been normal except for polydactyly in both hands and post-axial polydactyly of both feet. Her perinatal period was uncomplicated and she had been exclusively breastfed for six months and, subsequently, was commenced on formula milk and weaning from six months. Since then, she has had excessive weight gain and was investigated for obesity. Initial investigations had been normal, and she was managed on dietary modifications. Her extra digits in her hands had been resected at two years of age while polydactyly in her feet was not surgically corrected. Besides she had been prescribed spectacles for having poor vision at the age of three years. Her initial development had been normal but her preschool performance was below average.

**Figure 5:**
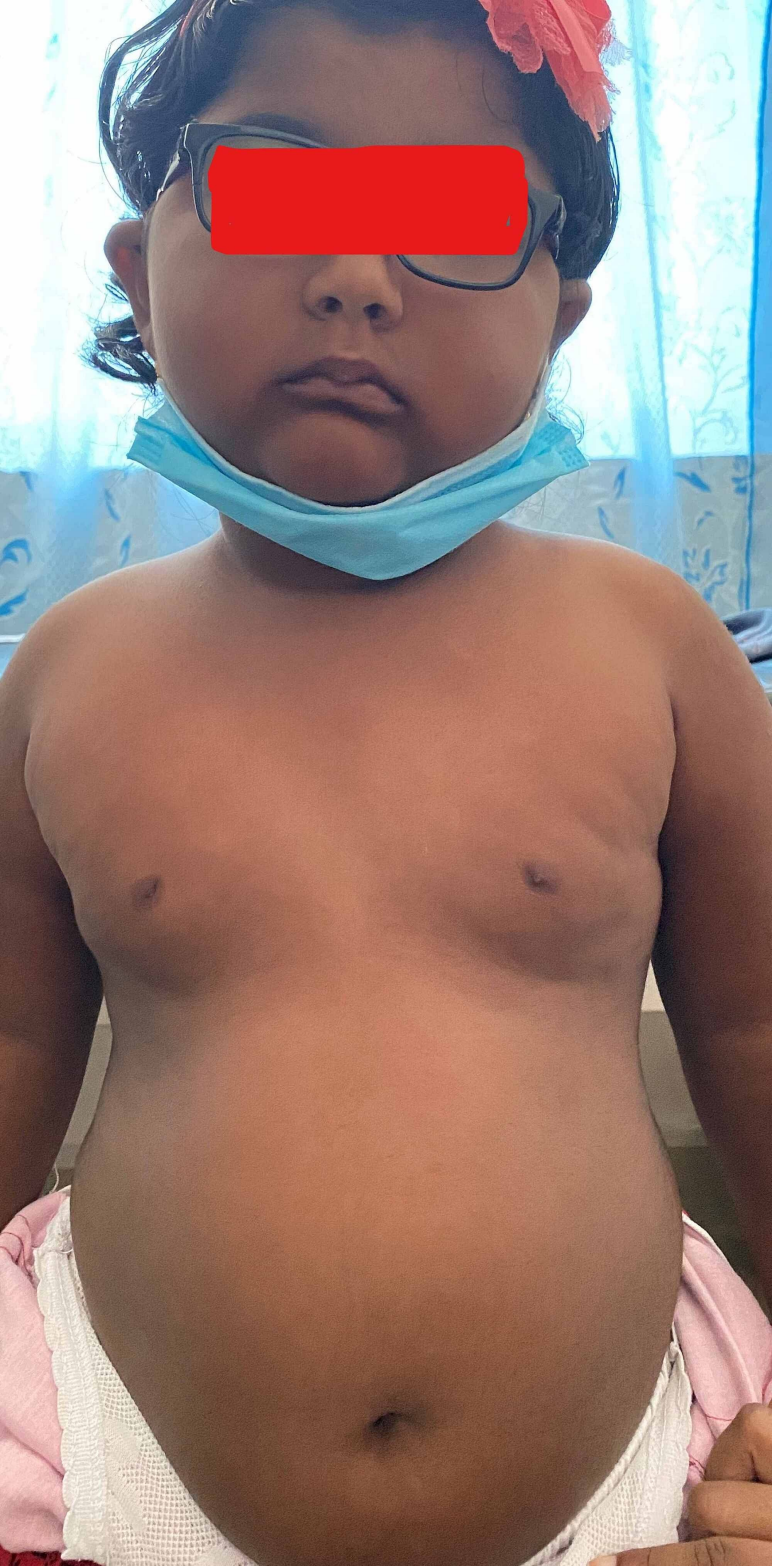
This obese girl shows a round face, enlarged breast, and pendulous abdomen

Physical examination showed a round face with puffy cheeks and acanthosis nigricans (Figure 5). Her weight was 24 kg (+2SD -+3SD); height was 101 cm (< (-) 1SD); and BMI was 23.5 kgm^2^ (>97th centile). She had short upper and lower limbs. Brachydactyly was present in both hands and feet with short, broad feet. There was a postaxial polydactyly in both feet and scar marks on both hands. Ophthalmological examination revealed visual acuity of 6/24 in both eyes with bilateral myopia and no features of retinitis pigmentosa. Gait was normal. His pulse rate was 100 bpm with good volume, all the peripheral pulses were palpable, and his blood pressure was 125/90 mmHg (both systolic and diastolic pressure was above 95th centile). There were no murmurs. The rest of the system examination was normal, including genitalia.

Laboratory investigations, including full blood count, renal function test, fasting blood sugar, liver function test, lipid profile, echocardiogram, and thyroid function, were normal. Other hormonal assays like growth hormone, cortisol, and norepinephrine were also normal. Renal biopsy revealed no abnormalities. Doppler scan of the renal artery excluded renal artery stenosis. Ultrasound abdomen revealed a renal cyst (17 × 7 mm) over the right kidney. Twenty-four hours of ambulatory blood pressure monitoring further confirmed the diagnosis of hypertension.

As the child had satisfied the criteria (Table 1), she was diagnosed with LMBBS with primary hypertension at four years of age at the tertiary care center. The patient was commenced with antihypertensive drugs and arranged for MDT clinic follow-up.

## Discussion

Bardet-Biedl syndrome (BBS) is the newest term used in place of the older LMBBS due to the phenotypic overlap between these conditions probably explained by the underlying allelic composition [9]. It is genetically heterogeneous with 21 BBS genes (BBS1-BBS20 and NPHP1) to date. The number can go up in the near future due to the discovery of whole-exome sequencing and ongoing analysis of unstudied populations. These BBS proteins are components of the centrosome that influence ciliary transport. This, in turn, perturbs ciliary function, and the syndrome is considered as a ciliopathy [3,10]. We did not do genetic studies on both reported children due to financial constraints in both families.

The expression of LMBBS differs in different children. The diagnostic criteria include both primary and secondary manifestations. The modified criteria define patients having either four or three primary criteria with two secondary criteria as having LMBBS [5,7-9]. As the disease phenotype has been variable and slowly evolving, the disease may not be diagnosed early [9]. Both these children satisfied the diagnostic criteria (Table 1) and were diagnosed late when they were referred for the management of hypertension.

Though retinal dystrophy is a common feature, the retinal appearance might vary, and typical retinitis pigmentosa appears in a smaller percentage of patients. The early identification of ophthalmological features, including strabismus, is mandatory to determine the visual prognosis, and the symptoms that appear within the first decade and poor night vision would be the first presentations. Conversely, children also present with poor vision and school performance [2,4,9]. Both these patients presented with difficulty in reading and both had strabismus and myopia for a long time, but the boy had early features of retinitis pigmentosa on last eye check-up. However, long-term follow-up in the ophthalmological clinic is recommended to identify the progress of retinitis pigmentosa.

Obesity is a well-known feature of LMBBS in childhood. The majority of children may have an average weight at birth, and, subsequently, develop obesity in infancy. It is known that the occurrence of obesity has been estimated in approximately 72% to 86% with an average of 75% [6,9]. The boy had a higher birth weight and continued to have accelerated growth since then. The girl had average weight at birth and accelerated growth from six months onward despite an average balanced diet.

Limb abnormalities had been estimated in varying frequencies [6,11]. Postaxial polydactyly, polydactyly, and brachydactyly of hands and feet have been common. Partial syndactyly, fifth finger clinodactyly, and a prominent gap between the first and second toes are also known to occur [6]. Both these children had postaxial polydactyly and brachydactyly at birth. Since extra digits are non-functional and create cosmetic problems, parents often remove them during infancy, similar to the second reported child. In children with both obesity and polydactyly that present in infancy, LMBBS merits consideration as a more favorable differential diagnosis [9].

Mental sub-normality is a borderline manifestation of this syndrome. An IQ of 79 or below is seen in 44% of patients of LMBBS, but only a minority of patients had mental subnormality according to the recent objective intelligence quotient (IQ) assessment [12]. Poor vision also contributed to low IQ [12]. Our index boy had learning problems to a greater extent while the girl had a below-average preschool performance. Both were referred for a special educational program.

Hypogonadism is known to occur in male children while females can have genital abnormalities such as hypoplastic fallopian tubes, uterus, ovaries, complete or partial vaginal atresia, absent vaginal orifices, or absent urethral orifice [12]. The index male child had a small penis with a pre-pubertal-sized testis and was confirmed to have hypogonadism and planned for hormonal treatment at 14 years, but the female child did not have obvious external abnormalities on examination and ultrasound abdomen.

There are several renal abnormalities detected in LBMMS, including chronic renal failure, parenchymal cyst, fetal lobulation, scarring, calyceal clubbing, unilateral agenesis, dysplastic kidneys, renal calculi, and vesicoureteral reflux. Chronic renal failure is the main cause of hypertension and morbidity in these patients [12]. Our index female child had a renal cyst, but neither of them had features of chronic renal failure.

Both of these patients had hypertension without any secondary causes. A child reported from Bangladesh with LMBBS also had hypertension [13]. Although it is not mentioned in the criteria, it may, therefore, be either a known finding in LMBBS or secondary to obesity.

## Conclusions

LMBBS is a complex genetic disease that has many functional and structural abnormalities. As there is no definite treatment, early diagnosis is essential to manage the complications related to this condition like retinitis pigmentosa, morbid obesity, and metabolic syndrome. Since it recurs in other children and carries a lot of morbidity, genetic study and genetic counseling are mandatory for patients who have a high index of suspicion based on prominent clinical findings such as childhood obesity, polydactyly, and consanguineous marriages.
